# Does arthroscopic repair show superiority over open repair of lateral ankle ligament for chronic lateral ankle instability: a systematic review and meta-analysis

**DOI:** 10.1186/s13018-020-01886-1

**Published:** 2020-08-26

**Authors:** Xiaosong Zhi, Zhuman Lv, Chen Zhang, Changwang Kong, Shijun Wei, Feng Xu

**Affiliations:** 1 Department of Orthopaedics, General Hospital of Central Theater Command (Wuhan General Hospital of Guangzhou Command, previously), No. 627, Wuluo Road, Wuhan, 430030 Hubei Province PR China; 2 Center for Stem Cells and Medicine, Department of Cell Biology, Navy Medical University (Second Military Medical University, previously), Shanghai, PR China

**Keywords:** Arthroscopic repair, Open repair, Lateral ankle ligament, Lateral ankle instability, Meta-analysis

## Abstract

**Background:**

There is still no definite consensus on whether arthroscopic repair shows superiority over open repair for chronic lateral ankle instability. We conducted a systematic review and meta-analysis of the current comparative studies to make a generalized analysis.

**Methods:**

PubMed, Embase, and Web of Science databases were searched from inception to April 2020. Included studies were assessed by the level of evidence and quality of evidence (Cochrane Handbook or MINORS). The process of data extraction was conducted by two independent authors. The comparative results of clinical outcomes, stress radiographic outcomes, and complication rates between two groups were pooled. Statistical analysis was performed using STATA.

**Results:**

Nine comparative studies for a total of 473 patients (250 arthroscopic repair, 223 open repair) were included. For the clinical outcomes, a significant difference was found in favor of arthroscopic repair with regard to AOFAS scores (MD 0.32, 95% CI 0.12 to 0.53, *I*^2^ = 7.7%, *P* = .370) and VAS scores (MD − 0.30, 95% CI − 0.54 to − 0.05, *I*^2^ = 48.3%, *P* = .102). No significant difference was found regarding to stress radiographic outcomes. Importantly, the total complication rate (RR 0.88, 95% CI 0.51 to 1.49, *I*^2^ = 0%, *P* = .957) as well as nerve complication rate (RR 1.21, 95% CI 0.53 to 2.75, *I*^2^ = 0%, *P* = .975) of arthroscopic repair group is not significantly different to that of open repair group.

**Conclusions:**

Arthroscopic repair for lateral ankle instability shows excellent clinical results comparable to open repair. Especially, arthroscopic repair might alleviate more pain due to the minimally invasive procedure. Patients receiving arthroscopic repair do not result in a higher total complication rate and nerve injury rate.

## Background

Ankle sprains are one of the most common injuries in activities/sports with a high recurrence rate, and repeated episodes could lead to chronic ankle instability, involving chronic insufficiency of the lateral ligament complex [1–3]. Among those, the anterior talofibular ligament (ATFL) is the most frequently affected ankle lateral ligamentfollowed by the calcaneofibular ligament (CFL) [4–7]. Conservative therapy is the initial treatment and is effective for major ankle sprains. However, there are still more than 20% of ankle sprains progressing into chronic lateral ankle instability and requiring operative treatments [8, 9].

The open modified Broström technique, first proposed in 1966, is an operative repair method for the lateral ankle ligaments when the ligament remnant is sufficient and the hindfoot alignment is good, and is widely regarded as the gold standard [10, 11]. This technique was modified by Gould in 1980 that reinforced the inferior extensor retinaculum (IER) after ATFL repair to strengthen ankle stability [12]. Recently, arthroscopic repair of the lateral ankle ligament, characterized by minimal invasiveness and fast recovery, has developed rapidly and has become increasingly popular [13, 14]. However, a high incidence of complications, especially postoperative nerve injury, is big trouble when arthroscopic repair is performed [15–18]. Up to date, there is inadequate clinical evidence in support of the use of arthroscopic repair or open repair techniques. In recent years, several clinical trials have aimed to compare arthroscopic and open repair for ATFL [11, 19–24]. These studies demonstrated some similar outcomes as well as different results between the two techniques after short- or mid-term follow-ups. However, there is still no definite consensus on which operative treatment shows overall or marked superiority over the other for chronic lateral ankle instability.

Our systematic review and meta-analysis aimed to comprehensively evaluate the current clinical evidence and statistically compare arthroscopic and open repair of lateral ankle ligament for chronic lateral ankle instability. We hypothesized that the current studies were in support of the arthroscopic repair technique concerning functional outcomes and complication rates when compared to the open repair technique.

## Materials and methods

### Literature search strategy

This study was conducted according to the guidelines outlined in the PRISMA (Preferred Reporting Items for Systematic Review and Meta-Analysis). PubMed, Embase, and Web of Science databases were searched to identify studies that reported the comparison between arthroscopic and traditional open procedures for chronic ankle instability up to April 2020. The search strategies were (Ankle instability OR Lateral ankle ligament OR talofibular) AND (Arthrosc* OR Endoscop*) AND (Minimally invasive OR open OR Broström). Additional studies were identified by secondary searches of reference lists of screened literature.

### Inclusion and exclusion criteria

The studies included should meet the following criteria: (1) a randomized controlled trial (RCT), prospective cohort, or retrospective cohort study design; (2) reporting the comparison between arthroscopic and traditional open technique for chronic ankle instability (no language restriction); and (3) providing the follow-up (at least 12 months) data.

An article was excluded based on the following criteria: (1) review articles, meta-analysis, case reports, editorial, technique articles, cadaveric studies or animal experiments; (2) unable to provide sufficient data, e.g., abstract from a meeting/conference; and (3) duplicated studies.

### Quality assessment

Each study was reviewed by two independent authors which graded the level of evidence [25]. Randomized studies were evaluated using the Cochrane Handbook (https://handbook-5-1.cochrane.org/). The risk of bias tool covers six items: selection bias, performance bias, detection bias, attrition bias, reporting bias, and other bias. Each item was categorized as “high,” “low,” or “unclear.” Non-randomized studies were assessed by methodological index for non-randomized studies (MINORS) containing 12 items with a global ideal score of 24 for comparative studies [26]. Any discrepancy was resolved by consulting an expert investigator until a consensus was reached.

### Data extraction

The data was extracted by two independent authors (Zhi X and Zhang C) based on inclusion and exclusion criteria mentioned above. The following information was collected: the last name of the first author, publication year, level of evidence, study design, publication country, number of patients, mean age, operative technique, follow-up time, operative time, clinical outcome, radiographic outcome, complication rate, and revision rate. Any disagreement was resolved by rechecking until a consensus was reached.

### Statistical analysis

Counting variables were analyzed with event number and total number. Continuous variables were analyzed with the weighted mean difference (MD). When a standard deviation of continuous variables was not reported, we contacted the corresponding author or calculated it through a method reported by Hozo et al. using the available data if possible [27]. Heterogeneity among studies was quantified using the *I*^2^ statistic [28]. *I*^2^ > 50% was considered to indicate substantial heterogeneity. When the *I*^2^ value was greater than 50%, the random effect model was applied. When *I*^2^ was less than 50%, the fixed effect model was used. STATA version 12.0 (StataCorp LP, College Station, TX, USA) was used for the whole meta-analysis. Statistical significance was set at *P* < .05.

## Results

### Literature search

**Fig. 1:**
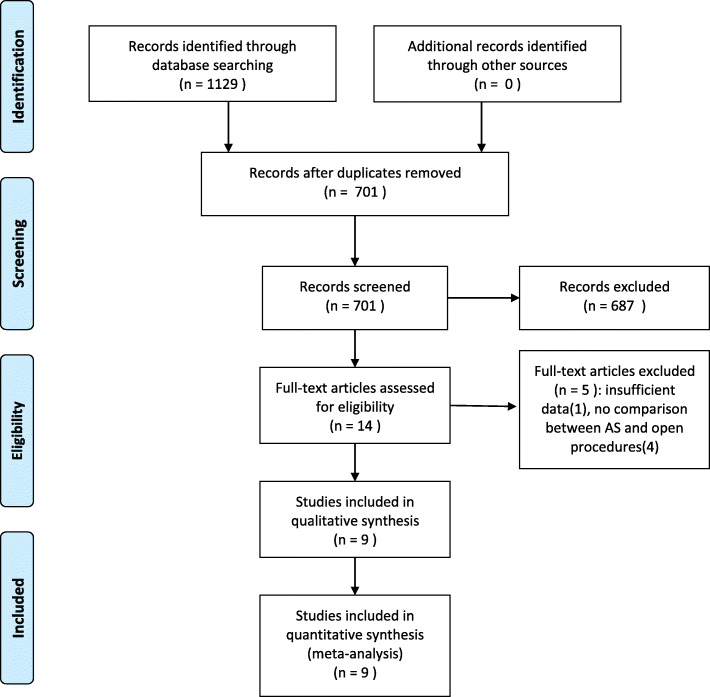
Flowchart of the literature selection

The electronic database search and additional search finally yielded 701 results after duplicate records were removed. The studies were screened by reviewing abstracts and full-text according to the inclusion and exclusion criteria, and nine studies were eligible for the final meta-analysis, including one randomized controlled trial (RCT), one prospective cohort study, and seven retrospective cohort studies. The studies were published from 2016 to 2020. Of those, one study was conducted in Republic of Korea, one in Japan, two in the USA, one in Singapore, and four in China. The detailed information is shown in Fig. 1 and Table 1. This study followed the PRISMA 2009 checklist as provided in Additional file 1.

**Table 1:** Characteristics of included studies

Study	Year	LOE	Type of study	Country	No. of patients		Age(years)	Operative technique	Follow-up (months)	Operative time
Yeo et al.	2016	1	RCT	Republic of Korea	48	AS 25	35.2	AS-assisted Broström–Gould, 1 suture anchors	12	2012–2014
						Open 23	34.3	Broström–Gould, 1 suture anchors	12	
Matsui et al.	2016	3	Retrospective cohort	Japan	37	AS 19	28	All-inside Broström–Gould, 2 suture anchors	12	2013–2014
						Open 18	24	Broström–Gould, 2 suture anchors	12	
Li et al.	2017	3	Prospective cohort	China	60	AS 23	30.3	All-inside Broström–Gould, 1/2 suture anchors	39.7	2012–2014
						Open 37	28.7	Broström–Gould, 1/2 suture anchors	35.5	
Rigby et al.	2018	3	Retrospective cohort	USA	62	AS 30	47.9	AS-assisted Broström–Gould, 2 suture anchors	15.6	2009–2013
						Open 32	37.7	Broström–Gould, 2 suture anchors	44.4	
DeVries et al.	2019	3	Retrospective cohort	USA	55	AS 43	44.7	All-AS Broström–Gould, 2 suture anchors	24.2	2014–2016
						Open 12	39.5	Broström–Gould, 2 suture anchors	21	
Yi et al.	2019	3	Retrospective cohort	China	65	AS 35	39.3	All-inside Broström–Gould, 1 suture anchors	26	2014–2017
						Open 30	37.3	Broström–Gould, 1 suture anchors	26	
Zeng et al.	2020	3	Retrospective cohort	China	27	AS 17	30.9	All-AS Broström–Gould, 1 suture anchors	36	2013-2015
						Open 10	27.7	Broström–Gould, 1 suture anchors	36	
Xu et al.	2020	3	Retrospective cohort	China	67	AS 32	33.7	Arthroscopic-assisted Broström, 1/2 suture anchors	36.5	2015–2017
						Open 35	35.8	Broström–Gould, 1/2 suture anchors	39.1	
Woo et al.	2020	3	Retrospective cohort	Singapore	52	AS 26	33.4	All-AS Broström–Gould, 2 suture anchors	12	2015–2019
						Open 26	31.5	Broström–Gould, 1 suture anchors	12	

*Abbreviations*: *LOE* level of evidence, *RCT* randomized controlled trial, *AS* arthroscopic

### Patient demographics

Four hundred seventy-three ankles were repaired in these 7 studies, of those, 250 with arthroscopic repair and 223 ankles with open procedures. The operations of all cases were performed in the time range of 2009–2019. All patients from the included studies received traditional open or arthroscopic Broström-Gould operations, with one or two suture anchors. The weighted mean age (years) of patients are 37.3 for the arthroscopic group and 33.3 for the open group. The overall weighted mean follow-up was 25.9 months (range 12–44.4 months). The weighted mean follow-up for cases with arthroscopic repair was 23.8 months (range 12–39.7 months), and that for cases with open procedures was 28.2 months (range 12–44.4 months). All patient demographics are summarized in Table 1.

### Quality assessment

According to established criteria, there was one study of LOE I and eight studies of LOE III. The study of Yeo et al. [11] was a RCT, so it was assessed by the Cochrane Handbook. The bias was assessed as selection bias (low), performance bias (low), detection bias (unclear, for inadequate information for the patients lost to follow-up), attrition bias (low), reporting bias (low), and other biases (low). The other studies were comparative non-randomized studies and were assessed by MINORS. All of the 8 non-randomized studies were scored above 17: Matsui et al. (17) [19], Li et al. (20) [20], Rigby et al. (18) [21], DeVries et al. (20) [22], Yi et al. (20) [23], Zeng et al. (18) [24], Xu et al. (22) [29], Woo et al. (19) [30].

### Clinical outcome

**Fig. 2:**
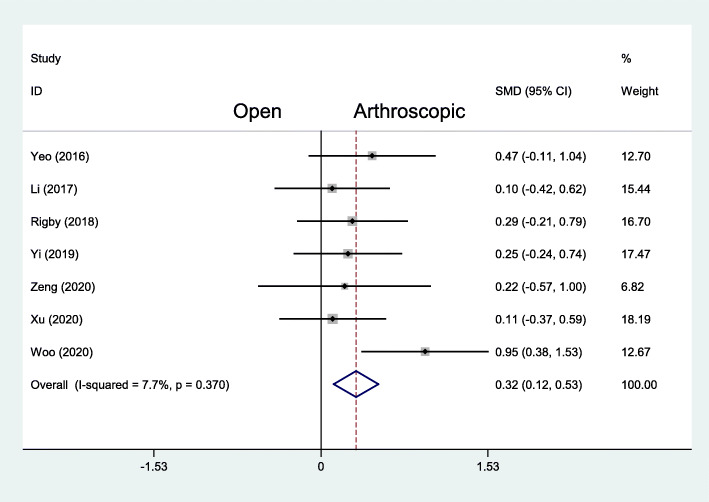
Forest plots of the comparison of arthroscopic and open technique for AOFAS score

The American Orthopaedic Foot & Ankle Society (AOFAS) score was given in 7 studies with 188 patients treated with the arthroscopic repair and 193 patients treated with the open repair. In the final follow-up evaluation, the average AOFAS score with the arthroscopic repair was 92.3, and the average AOFAS score with the open repair was 90.8. There was a statistically significant difference in favor of the arthroscopic repair (MD 0.32, 95% CI 0.12 to 0.53, *I*^2^ = 7.7%, *P* = .370) (Fig. 2). For short-term outcome, Yi et al. reported that AOFAS score at 2 weeks after operation in the arthroscopic group was 79.5 ± 6.4, significantly higher than that score in the open group that was 76.3 ± 4.9 (*t* = 2.234, *P* = .029) [23].

**Fig. 3:**
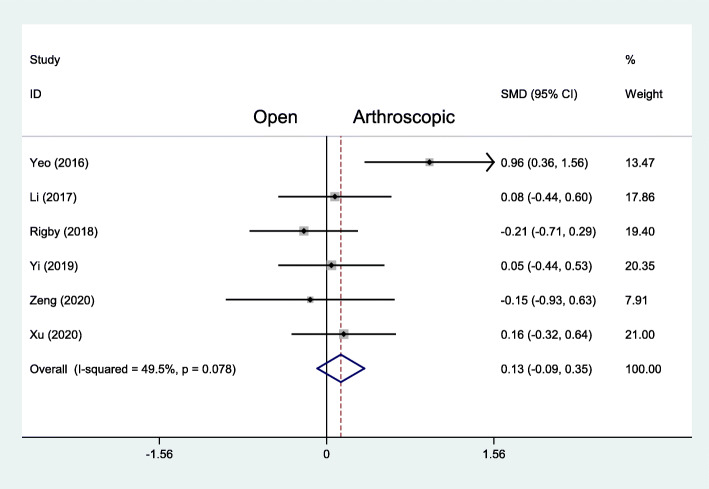
Forest plots of the comparison of arthroscopic and open technique for Karlsson score

The Karlsson score was reported in 6 studies with 162 patients treated with the arthroscopic repair and 167 patients with the open repair. In the final follow-up evaluation, the average Karlsson score with the arthroscopic repair was 86.6, and with the open repair the mean value was 86.3. There was no statistically significant difference (MD 0.13, 95% CI − 0.09 to 0.35, *I*^2^ = 49.5%, *P* = .078) (Fig. 3). But for the short-term results from Yi et al., Karlsson score at 2 weeks after operation in the arthroscopic group was 77.2 ± 8.8, significantly higher than in the open group that was 73.4 ± 5.8 (*t* = 2.065, *P* = .043) [23].

**Fig. 4:**
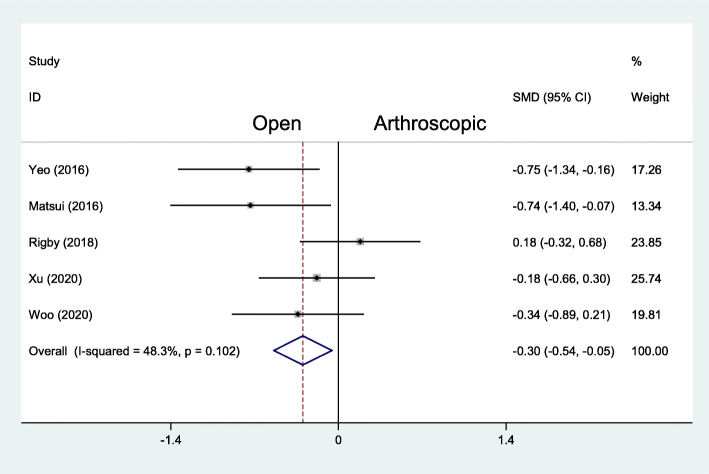
Forest plots of the comparison of arthroscopic and open technique for VAS score

The visual analog scale (VAS) score for pain evaluation was reported in 5 studies with 132 patients treated with the arthroscopic repair and 134 patients treated with the open repair. In the final follow-up evaluation, the average VAS score with the arthroscopic repair was 1.51, significantly lower than 1.84 in the open repair group (MD − 0.30, 95% CI − 0.54 to − 0.05, *I*^2^ = 48.3%, *P* = .102) (Fig. 4). According to the studies of Yeo et al. [11] and Matsui et al. [19], the VAS score of both groups were not significantly different at short-term follow-ups (2 weeks and 6 weeks).

Two studies [19, 23] reported with the Japanese Society for Surgery of the foot ankle-hindfoot (JSSF) scale score with 54 patients being treated with the arthroscopic repair and 48 patients being treated with the open repair. Yi et al. [23] reported that JSSF score at 2 weeks after operation in arthroscopic group was 87.8 ± 8.4 and that in the open group was 83.5 ± 7.6 with significant difference (*t* = 2.149, *P* = .035). Whereas, there was no statistically significant difference when JSSF score was assessed at 3 months [23], and at the final follow-ups [19, 23] after operation.

### Stress radiographic outcome

**Fig. 5:**
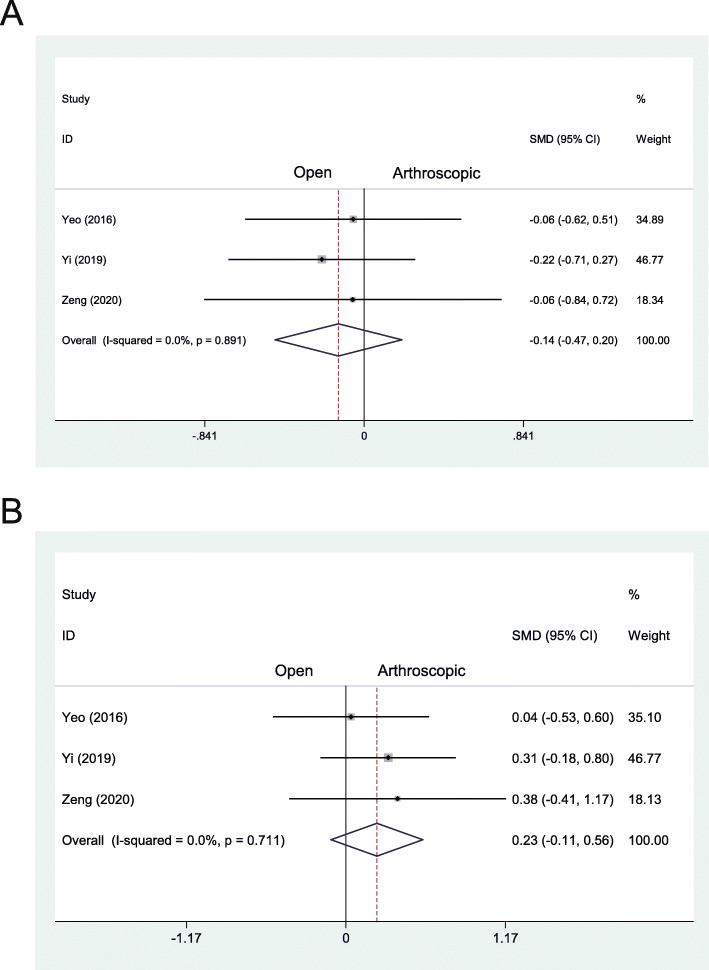
**a** Forest plots of the comparison of arthroscopic and open technique for anterior displacement of talus at final follow-up. **b** Forest plots of the comparison of arthroscopic and open technique for tilt angle of talus at final follow-up

In the pooled results from Yeo et al., Yi et al., and Zeng et al., no significant difference were found in postoperative anterior displacement (MD − 0.14, 95% CI − 0.47 to 0.20, *I*^2^ = 0.0%, *P* = .891) (Fig. 5a) or tilt angle of the talus between the two groups (MD 0.23, 95% CI − 0.11 to 0.56, *I*^2^ = 0.0%, *P* = .771) (Fig. 5b). In the studies of Yeo et al. [11] and Zeng et al. [24], stress radiographic data revealed no significant difference of change (preoperative to postoperative) of either anterior displacement or tilt angle of the talus between the two groups. In the studies of Matsui et al. [19] and Yi et al. [23], no significant difference existed in preoperative or postoperative anterior displacement of the talus between the two groups. Additionally, there was also no significant difference when comparing the tilt angle of the talus between the two groups [23]. The remaining studies did not report the data of the stress radiographic outcomes.

### Complications and revisions

**Fig. 6:**
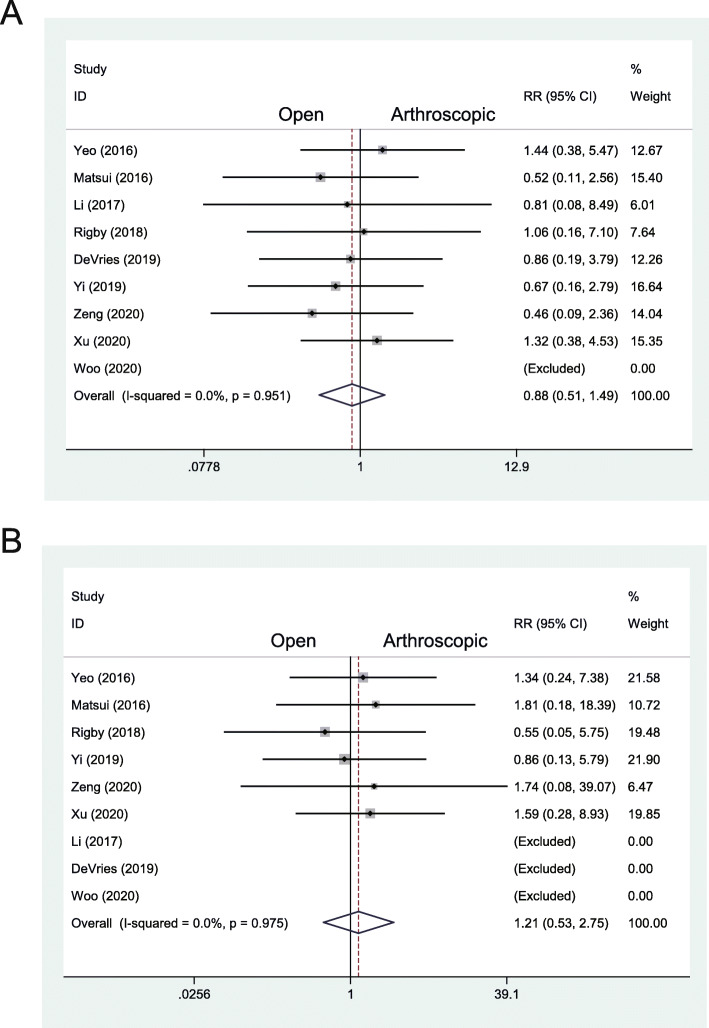
**a** Forest plots of the comparison of arthroscopic and open technique for total complication rate. **b** Forest plots of the comparison of arthroscopic and open technique for nerve complication rate

The complications were reported in 9 studies with 250 patients treated with the arthroscopic repair, including 12 had nerve injuries, 5 had knot pain, 1 had persistent pain, 1 had deep venous thrombosis (DVT), 1 had an infection, 1 suffered from poor healing, and 5 had revisions (Table 2). In 223 patients treated with the open repair, there were 9 nerve injuries, 4 infections, 3 wound irritations, 2 had persistent pain, 1 had tendinitis, 3 had knot pain, and 2 suffered from poor healing (Table 2). None of the patients were reported to receive a revision surgery in the open group (Table 2). Arthroscopic cases had a total complication rate of 10.4%, whereas the total complication rate of the open repair was 10.8%. There was no statistically significant difference between the two techniques with regard to the overall complication rate (RR 0.88, 95% CI 0.51 to 1.49, *I*^2^ = 0%, *P* = .957) (Fig. 6a). Then we compared the nerve complication rate between the two groups additionally. The results showed that no statistically significant difference existed between the two techniques concerning the nerve complication rate (RR 1.21, 95% CI 0.53 to 2.75, *I*^2^ = 0%, *P* = .975) (Fig. 6b).

**Table 2:** Outcomes and complications in identified studies

Study	Group	AOFAS	Karlsson	VAS	JSSF	Anterior drawer test (mm)	Talar tilt angle (°)	Complication rate	Revision rate
Yeo et al.	AS	90.3	76.2	1.7	NR	− 1.7(8.4 to 6.7)	− 3.4(7.3 to 3.9)	20%(3:nerve injury, 2:knot pain)	NR
	open	89.2	73.5	2	NR	− 1(7.8 to 6.8)	− 1.6(5.4 to 3.8)	13%(2:nerve injury, 1:infection)	NR
Matsui et al.	AS	NR	NR	1.2	98	− 5.6(8.4 to 2.8)	− 6.8(10 to 3.2)	10.5%(2:nerve injury)	0%
	open	NR	NR	1.9	95.4	− 6.2(9.1 to 2.9)	− 7(9.9 to 2.9)	22.2%(3:wound irritation, 1:nerve injury)	0%
Li et al.	AS	93.3	90.3	NR	NR	NR	NR	4.3%(1:persistent pain)	0%
	open	92.4	89.4	NR	NR	NR	NR	5.4%(2:persistent pain)	0%
Rigby et al.	AS	95.33	91.8	1.5	NR	NR	NR	6.7%(1:nerve injury, 1:DVT)	NR
	open	93.53	93.41	1.2	NR	NR	NR	6.3%(2:nerve injury)	NR
DeVries et al.	AS	NR	NR	NR	NR	NR	NR	14%(5:revision, 1:infection)	11.6%
	open	NR	NR	NR	NR	NR	NR	16.7%(1:tendinitis, 1:infection)	0%
Yi et al.	AS	93.4	89.3	NR	97.2	− 5.4(8.6 to 3.2)	− 5.5(8.8 to 3.3)	8.6%(2:nerve injury, 1:knot pain)	0%
	open	91.8	88.9	NR	95.6	− 5.6(8.9 to 3.3)	− 6.4(9.5 to 3.1)	13.3%(2:nerve injury, 2:knot pain)	0%
Zeng et al.	AS	92.4	89.2	NR	NR	− 2.8(12.9 to 10.1)	− 2.4(11.2 to 8.8)	11.8%(1:nerve injury, 1:poor healing)	NR
	open	91.1	90.5	NR	NR	− 3.3(13.6 to 10.3)	− 2.7(10.4 to 7.7)	30%(1:knot pain, 2:poor healing)	NR
Xu et al.	AS	87.7	NR	1.8	NR	NR	NR	15.6%(3:nerve injury, 2:knot pain)	NR
	open	86.9	NR	2.1	NR	NR	NR	11.4%(2:nerve injury, 2:infection)	NR
Woo et al.	AS	94.2	NR	1.2	NR	NR	NR	0.0%	0%
	open	70.9	NR	2.1	NR	NR	NR	0.0%	0%

*Abbreviations*: *AOFAS* American Orthopaedic Foot and Ankle Society, *VAS* visual analog scale; *JSSF* Japanese Society for Surgery of the foot ankle-hindfoot, *AS* arthroscopic, *NR* not reported, *DVT* deep venous thrombosis

### Return to full activity/sports

Matsui et al. [19] reported that the mean time to return to daily activity was significantly shorter in the arthroscopic group: 5.3 (range 3–12) weeks vs 7.1 (range 5–12) weeks in the open group (*P* < .05). However, there was no significant difference in mean time to return to activity/sports: 16.5 (range 12–22) weeks in the arthroscopic group vs 17.1 (range 13–22) weeks in the open group (*P* = .07). Yi et al. [23] reported that the length of time to recover from surgery was 5.4 ± 1.7 weeks in the arthroscopic group, and that time was 7.2 ± 2.3 weeks in the open group, with significant difference (*t* = 3.620, *P* = .001). Rigby et al. [21] reported that the rate of return to sports activity was 96.7% (29 of 30 patients) in the arthroscopic group and 96.9% (31 of 32 patients) in the open group. DeVries et al. [22] showed that the 35 out of 43 patients returned to activity/sports in the arthroscopic group, the returning time was 127.2 ± 96.3 (range 53–569) days. Nine out of 12 patients returned to activity/sports in the open group, the returning time was 170.7 ± 66.4 (range 56–174) days. There was a statistically significant difference between the groups with regard to the time other than the rate to return to activity/sports (*P* = .008). The remaining studies did not report time of surgery recovery and returning to activity/sports.

## Discussion

The highlight of this study was that arthroscopic repair of ATFL is a very safe and alternative technique for restoring lateral ankle instability. The comprehensive results suggest that arthroscopic repair of ATFL may result in superior clinical outcomes (AOFAS and VAS score) over open ATFL repair, although no significant difference is found for the stress radiographic outcomes between the two techniques. Additionally, the total complication rate and nerve complication rate were shown to be similar between the two procedures.

The surgical treatment for ATFL injury includes ATFL repair and ATFL reconstructive technique (preservation of ATFL or no preservation of ATFL) [31]. The open Broström-Gould procedure is widely accepted as the gold standard technique for lateral ankle ligament repair [32]. The technique is one method of anatomical repair of the ATFL with many advantages, e.g., low technical complexity, low incidence of complications, allowing subtalar motion and showing good clinical effectiveness [10, 33]. Arthroscopic ankle stabilization was first described by Hawkins [13] by using a staple technique and developed by Ferkel and Scranton [34] in the 1990s. But arthroscopic repair did not gain popularity in that time due to higher complexity, longer operative time, and more complications compared to conventional open repair [18, 24]. Kashuk et al. firstly performed an arthroscopic technique using suture anchors for lateral ligamentous complex repair, which offered the benefit of quicker recovery [35]. With the rapid development of arthroscopic instruments and the simplification of arthroscopic repair techniques, the advantages of minimally invasive arthroscopy have become more apparent [24]. Besides, other intra-articular lesions can be simultaneously treated during arthroscopic procedures [36]. However, the complication rate remains relatively high [37]. Recent nine studies respectively aimed to find the evidence-based advantages of either technique by comparing the arthroscopic repair and conventional open procedure in clinical trials. However, individual studies show a low level of evidence, so there is still no clear consensus on which operative treatment possesses superiority over the other for chronic lateral ankle instability. Thus, we performed this meta-analysis of the nine comparative studies to achieve a comprehensive evaluation.

We found that the arthroscopic repair showed an overall better AOFAS score than the open method in final follow-ups. However, there is no significant difference between the two groups when comparing the Karlsson-Peterson scores in final follow-ups. These results were similar to the previous meta-analysis by Brown et al. [38]. Interestingly, we also compared the VAS scale between the two groups, and from the pooled results, we found that arthroscopic repair might alleviate more pain due to the minimally invasiveness of this procedure. When it comes to early recovery, AOFAS scores, Karlsson scores, and JSSF scores at 2 weeks after surgery seem to be in support of arthroscopic repair [23]. However, Yeo et al. reported that there was no significant difference between the two groups when AOFAS scores, Karlsson scores, and VAS scores were evaluated at 6 weeks after surgery [11]. Actually, in our clinical work, we found that the AOFAS score was not the best evaluation scale for ankle instability and the Karlsson score was more effective.

With regard to the stress radiographic outcomes, the talar tilt angle and anterior displacement of the talus are the most common index for evaluation. From the included studies, no matter for the preoperative comparison, postoperative comparison, or for the change (preoperative to postoperative) of the talar tilt angle and anterior displacement of the talus, radiographic outcomes did not show significant difference between the arthroscopic group and the open group. However, whether stress radiographs is indispensable for diagnosis or assessing clinical outcomes remains debatable [39, 40]. Lee et al. [41] explained that the results of the stress radiographs were not so reliable because of the possibility of false-negative errors in case of muscle contraction. Furthermore, Hertel et al. [42] reported that patients with functional ankle instability showed normal stress radiographic outcomes. MRI is a valuable diagnosing method for evaluating intra-articular pathology, but it is more used to find abnormality of the lateral ankle ligament as well as concomitant injury rather than to evaluate the clinical outcomes after surgery [43].

Although a previous systematic review reported that the arthroscopic technique had a complication rate of 15.27%, higher than that of 7.92% in the open repair [18], from the pooled results of our study, there was no statistically significant difference between the two groups (10.4% for the arthroscopic repair, 10.8% for the open repair). One of the most common complications is the occurrence of nerve injury, especially during arthroscopic repair procedures [44]. The arthroscopic techniques include arthroscopic-assisted techniques, all-arthroscopic techniques, and all-inside techniques [45]. Among those, suture knots of all-inside techniques are tied within the joint, while sutures are tied extracapsularly in the other two techniques, which increase the risk of entrapment of surrounding anatomical structures and increase the complicate rate [46]. In order to reduce the incidence of nerve injury during arthroscopic repair, Acevedo et al. identified a “safe zone,” with a mean length of 51 mm between the intermediate branch of the superficial peroneal nerve (SPN) and sural nerve, and found that a safe distance of 20 mm (range, 8–36 mm) was maintained from the most medial suture to the intermediate branch of the SPN [47]. With the simplification of arthroscopic technique and repeated training of surgeons, the incidence of nerve injuries is becoming much lower now than before [19, 20, 47, 48]. In this meta-analysis, the complication rate of nerve injuries is 4.69% in the arthroscopic repair, and 4.32% in the open group, also with no statistically significant difference.

A systematic review reported by So et al. included 11 studies involving 669 cases receiving the open Broström-Gould procedures, and the revision rate was 1.2% at a weighted mean follow-up of 8.4 years [49]. A prospective study by Lopes et al. of 286 patients with the arthroscopic repair or reconstruction reported a 4.2% revision rate during mean follow-up of 9.6 months [50]. Up to date, there are rare studies comparing revision rate between the two techniques. In this review, 5 of the 9 studies reported a 0% revision rate in the open repair, and the other 4 studies did not report this data. For the arthroscopic repair, 4 of the 9 studies reported a 0% revision rate, whereas, one study reported an 11.6% revision rate, much higher than the other studies. So, the current evidence for revision of arthroscopic repair is still limited and further studies with larger amounts of patients are necessary.

Returning to activity/sports indicated a mid- to long-term recovery from ankle instability. In this review, Rigby et al. [21] found that the rates of returning to sports in the two groups were both 97%, and Matsui et al. [19] stated that no significant difference existed between the two groups with regard to the mean time of returning to sports. Only DeVries et al. [22] found that the returning time to activity/sports in the arthroscopic group was shorter than that in the open group, but the returning rate showed no significant difference. It is noteworthy that faster recovery and earlier return to activity/sports may rely not only on operative techniques but also on postoperative management. A meta-analysis reported that patients with early mobilization (within 3 weeks after surgery) postoperative protocols demonstrated improved functional outcomes [51]. Postoperative management, especially the time to allow weightbearing, varied in these studies. Matsui et al. [19] reported that weightbearing and active range of motion exercise were permitted the day after surgery. Patients of Yeo et al. [11], DeVries et al. [22], Yi et al. [23], and Zeng et al. [24] remained non-weightbearing until 2 weeks. Li et al. [20] reported that weightbearing was permitted after 4 weeks. In the study of Rigby et al. [21], they allowed full weight bear after 20 days in the open group, whereas the time in the arthroscopic group was 3 days because of their hypothesis that patients had less swelling and pain. Thus, there is still no consensus on the proper time to allow weightbearing up to date.

There are also several limitations and potential biases to pay attention to. Eight included studies were LOE III, and only one was RCT of LOE I. The follow-up time: (1) varied among these studies; (2) less than 2 years in four studies; (3) within one study, the time length in the arthroscopic group was far less than that of the open group. Besides, intra-articular comorbidity existed in patients and was treated during operative procedures in seven studies. However, the patients with additional pathology in the other two studies were excluded. With regard to the data collection, there was no clear statement whether surgeons and investigators were separated to assess the outcomes in the included studies.

## Conclusion

In conclusion, arthroscopic repair for lateral ankle instability shows excellent clinical results comparable to open repair. Patients receiving either procedure result in similar functional and radiographic outcomes with equivalent complication rates. Especially, arthroscopic repair, characterized by minimal invasiveness, might provide better recovery (AOFAS scores) and alleviate more pain (VAS scores). Further RCTs with a larger sample-size and longer follow-up time are still required to achieve a more reliable conclusion.

## Supplementary information

**Additional file 1.** PRISMA 2009 checklist

## Data Availability

The datasets used and/or analyzed during the current study are available from the corresponding author on reasonable request.

## Abbreviations

ATFL: Anterior talofibular ligament
CFL: Calcaneofibular ligament
IER: Inferior extensor retinaculum
RCT: Randomized controlled trial
MINORS: Methodological index for non-randomized studies
MD: Mean difference
AOFAS: American Orthopaedic Foot & Ankle Society
VAS: Visual analog scale
SPN: Superficial peroneal nerve
LOE: Level of evidence
AS: Arthroscopic
JSSF: Japanese Society for Surgery of the foot ankle-hindfoot
NR: Not reported
DVT: Deep venous thrombosis
